# Glymphatics for the Neurosurgeon

**DOI:** 10.1227/neuprac.0000000000000051

**Published:** 2023-07-11

**Authors:** Randall W. Treffy, Akram M. Eraky, Omar Hussain, Hirad S. Hedayat

**Affiliations:** Department of Neurosurgery, Medical College of Wisconsin, Milwaukee, Wisconsin, USA

**Keywords:** Glymphatics, Cerebrospinal fluid, Virchow-Robbin spaces, Traumatic brain injury, Normal pressure hydrocephalus

## Abstract

The glymphatic system is a recently described open vascular system within the brain that allows cerebrospinal fluid to flow into brain parenchyma through perivascular spaces and clears interstitial solutes into the traditional closed vascular system. Although it was initially difficult to study, new evidence suggests that the glymphatic system plays a pivotal role in the pathophysiology of multiple diseases of the central nervous system including traumatic brain injury, hydrocephalus, dementia, and malignancy. In this review, we cover the basics of cerebrospinal fluid dynamics and explore the role of the glymphatic system in traumatic brain injury, post subarachnoid hemorrhage hydrocephalus, normal pressure hydrocephalus, and malignancy. We believe that further understanding of the glymphatic system will lead to new and better therapeutic options for these diseases moving forward.

**ABBREVIATIONS:**
AQP4: aquaporin-4
ISF: interstitial fluid
LD: lumbar drain
NPH: normal pressure hydrocephalus
TBI: traumatic brain injury.

Cerebrospinal fluid (CSF) is 1 of the 3 main components of the intracranial space along with the brain parenchyma and blood.^1^ Although clearly an important component of the intracranial space, there is much controversy regarding its production, resorption, and its role in central nervous system homeostasis.^2^

CSF production was classically thought to be derived from the choroid plexus, an area of highly vascular tissue within the ventricles and was absorbed along arachnoid granulations in the dural sinuses with a production rate around 0.3–0.4 cc's a minute and a total volume of 90–150 cc's in an adult human.^3-7^ Although the classic theories of CSF production, flow, and resorption were helpful in understanding many diseases of the brain, including hydrocephalus, they fail to fully explain the complete spectrum of CSF-driven central nervous system pathologies.^1-3^

## GLYMPHATIC SYSTEM

Throughout the rest of the body, there exists a secondary circulatory system, known as the lymphatic system, which returns interstitial fluid (ISF) and macromolecules to the circulation, and aids in immune surveillance reviewed extensively by Choi et al.^8^ This system is critical to tissue homeostasis as the “closed” circulatory system leaks fluid into the interstitial space, and without a return mechanism, edema occurs. Despite having a high metabolic rate, the brain was classically thought to lack a lymphatic system; it was thought that perhaps CSF serves as a lymphatic system, and studies had shown bulk flow of molecules out of ISF into CSF.^9^

**FIGURE 1.**
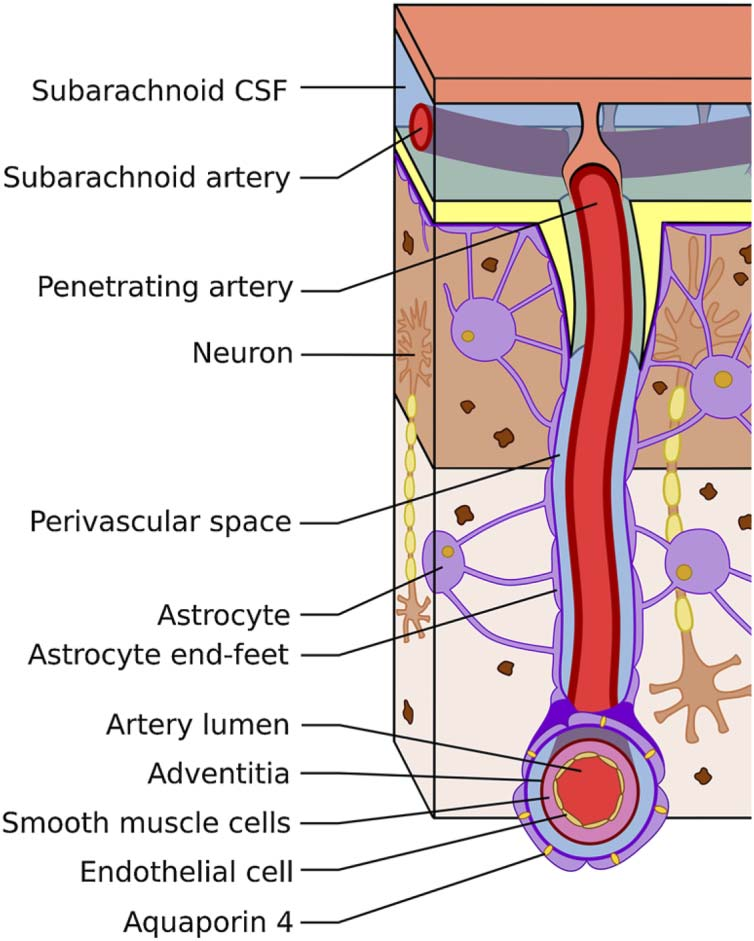
An illustration showing a cortical artery surrounded by the Virchow-Robin space; reproduced from Ramirez et al,^47^ with permission from Springer Nature. The Virchow-Robin space is a perivascular space passing through the subarachnoid and subpial spaces to the brain parenchyma. Virchow-Robin spaces surround arteries, arterioles, veins, and venules and are thought to be the area at which CSF passes into brain parenchymal interstitial spaces. CSF, cerebrospinal fluid.

**FIGURE 2.**
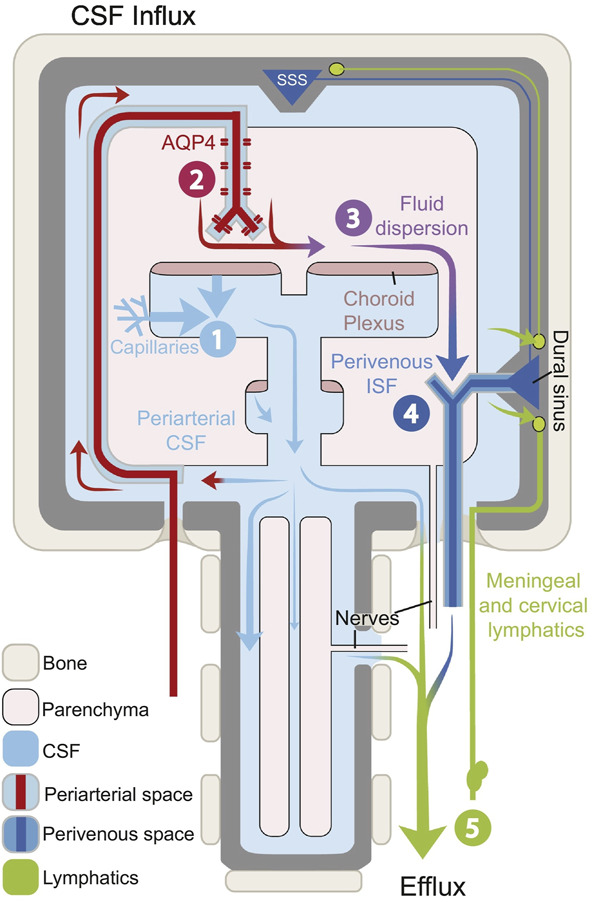
Schematic model of the glymphatic system; reproduced from Mestre et al^2^ under the Creative Commons Attribution – NonCommercial – NoDerivs (CC BY-NC-ND 4.0) license: CSF is first produced by the choroid plexus mostly within the ventricular system (1). CSF then flows through the ventricular system and into the subarachnoid spaces, eventually making its way to the basal cisterns. Once CSF has reached the basal cisterns, arterial pulsation drives CSF into Virchow-Robin spaces (2) into the brain parenchyma. At this point, the CSF and ISF mix with the assistance of AQP4 on glial footplates (3). The newly mixed CSF and ISF then drain via perivenous pathways (4) into the dural sinuses or via dural and meningeal lymphatics (5) as well as along cranial nerves. AQP4, aquaporin-4; CSF, cerebrospinal fluid; ISF, interstitial fluid; SSS, superior sagittal sinus.

Iliff et al^10^ injected tracer molecules into the ventricular system of mice and observed minimal diffusion into the brain other than the nearby ependymal lining. They then went on to inject tracer molecules into the subarachnoid space and demonstrated that smaller molecules would diffuse into the brain parenchyma while larger molecules would spread into perivascular spaces (Virchow-Robin spaces; Figure 1) and slowly diffuse into the brain parenchyma.^10^ Using timed experiments, they discovered that the tracers would accumulate along arterial perivascular spaces first and would be devoid along venous perivascular spaces. However, as time progressed, the tracers would be seen along the venous pathways; tracer would also drain along venous pathways when injected directly into brain parenchyma.^10^ It was thought that CSF was pushed through the perivascular spaces by arterial pulsations and was further demonstrated in studies using MRI in living animals in which middle cerebral artery distribution strokes obstructed this pathway.^11^ These findings demonstrated that both CSF and ISF drained through a perivenous pathway and eventually ended up in cervical lymphatics. They then went on to demonstrate that aquaporin-4 (AQP4) was required for their observation of CSF/ISF drainage along perivenous pathways.^10^ This newly discovered network was termed the glymphatic system because it relies on glial water efflux and acts as a lymphatic system. Its description has led to a new understanding of the fluid dynamics of the brain (Figure 2; reproduced with permission by Mestre et al^2^).

Shortly after the discovery of the glymphatic system, additional discoveries were made regarding the outflow of CSF and ISF from the brain. Although most CSF/ISF from the glymphatic system was likely resorbed into the venous system, there was still past findings of proteins injected into CSF found in cervical lymph nodes that were not explained by this system.^12^ It was previously thought that the system of drainage leading to proteins from CSF being found in lymph nodes was likely drainage of CSF through cranial nerves, most likely from the cribriform plate, optic nerve, and trigeminal nerve.^12^

Using mice with reporters known to mark lymphatic vessels, Aspelund et al^13^ discovered an extensive network of lymphatic vessels along the dura. These lymphatic vessels were associated with cranial nerves, meningeal blood vessels, and venous drainage suggesting that lymphatic vessels exit the skull through the conventional foramina.^13^ They then went on to find that tracers injected into brain parenchyma drained through the glymphatic system with some drainage also into the described meningeal lymphatics and then into the cervical lymph nodes.^13^ In mice whose meningeal lymphatics have been removed by genetic perturbation, overall fluid volume remained unchanged within the brain. The ability to transport macromolecules appeared impaired suggesting that the role of the meningeal lymphatics is assisting in draining of large macromolecules.^13^ These new findings of the glymphatic system coupled with meningeal lymphatics have opened the possibility for a new realm of therapeutic interventions.

## TRAUMATIC BRAIN INJURY

Traumatic brain injury (TBI) remains a major source of morbidity and mortality throughout the world.^14^ Given this high morbidity and mortality, there is much interest in the optimal treatment for such injuries. Although the primary insult may lead to significant neurological injury, secondary injury due to swelling occurs and is a major source of the morbidity and mortality of those who survive the initial insult.^15^

The Monro-Kellie doctrine explains that given the rigid nature of the skull, there is only a fixed amount of volume for the 3 major intracranial components: brain parenchyma, blood, and CSF.^1^ Swelling may cause significant disruption in the delicate balance between these 3 components, causing compression of brain parenchyma, leading to direct injury or ischemia because of decreased cerebral perfusion.

If medical management is not enough to control intracranial pressure (ICP), surgical options are pursued which include external ventricular drain, craniotomy, or decompressive craniectomy.^15^ Two major studies have compared maximal medical management vs maximal medical management plus decompressive craniectomy in the treatment of patients with severe TBI. Decompressive Craniectomy in Diffuse Traumatic Brain Injury (DECRA) studied the role of decompressive craniectomy for medically refractory ICPs and found that decompressive craniectomy was inferior to maximal medical management alone.^16^ This study was limited and did not involve patients with mass lesions such as subdural hematoma or intraparenchymal hemorrhage and used bifrontal decompressive craniectomy only. Randomized Evaluation of Surgery with Craniectomy for Uncontrollable Elevation of Intracranial Pressure also compared the role of decompressive craniectomy vs maximal medical management in the role of refractory ICP but also allowed the inclusion of mass lesions. Randomized Evaluation of Surgery with Craniectomy for Uncontrollable Elevation of Intracranial Pressure found that decompressive craniectomy (hemicraniectomy or bifrontal decompressive craniectomy) improved survival when compared with maximal medical management, but there was no significant difference in the number of patients with favorable outcomes.^17^ These results indicate that there exists a need for better surgical interventions.

In addition to craniectomy, another technique to decrease ICP is CSF diversion which results in increased space for brain parenchyma and blood.^15^ The major technique for CSF diversion is external ventricular drain placement in the lateral ventricle. Given the findings above that suggest that ventricular CSF does not communicate directly with brain parenchyma, but that CSF within the basal cisterns does,^18^ a new theory suggests a portion of post-traumatic brain edema may be caused by CSF egress into interstitial spaces within brain parenchyma. Elevated pressure because of poor CSF resorption causes CSF to flow into the relatively lower pressure interstitial space within the brain parenchyma leading to cerebral edema. It could then be inferred that drainage from the basal cisterns may, therefore, relieve this edema and may lead to decreased brain swelling, decreased herniation out of the cranial defect, and may decrease potential axonal stretch injury that may occur with significant brain herniation.^18^

Given this finding, it was surmised that microsurgically opening the basal cisterns with external drainage (cisternostomy) may improve outcomes in patients. A single surgeon in South Asia described this procedure and from his studies found what appeared to be improved outcomes, improved survival, decreased time on the ventilator, and improved 6 week Glasgow Outcome Score.^19^ A single center added cisternostomy as an adjuvant to their decompressive hemicraniectomies and in their limited study found a nonsignificant increase in positive outcomes (Glasgow Outcome Score Extended greater than 5) (61.1% with cisternostomy compared with 35% without cisternostomy *P* = .1).^20^ More recently, another center performed a randomized control trial of 50 patients (25 in each group) comparing cisternostomy and decompressive craniectomy and found improved ICP as well as some improved outcomes in select groups.^21^ Although these results are promising, they are from small groups of patients and a limited number of surgeons, and additional studies will need to be performed to further validate this procedure. In addition, cisternostomy can be a difficult procedure to perform, especially in the setting of significant swelling secondary to traumatic injury.

Some evidence to potentially support the benefit of drainage of cisternal CSF is the use of lumbar drains in TBI. A lumbar drain is placed within the lumbar cistern allowing continuous drainage of CSF that communicates with the basal cisterns. Although lumbar drains have traditionally been avoided because of concern for worsening tentorial herniation, a recent systematic review demonstrated that in TBIs, there is at least a significant decrease in ICP after placement.^22^ The studies included in this systematic review are small, the data are mixed, and it is unclear whether there is a significant change in outcomes. However, these results are interesting and suggest a potential role of cisternal drainage in TBI.

In addition to the glymphatic system, the meningeal lymphatics also play a role in TBI. Bolte et al^23^ discovered that in their mouse model of TBI, there was significant disruption in the meningeal lymphatics which continued up to a month after the TBI. This disruption altered the drainage of both macromolecules and CSF. One of the causes of lymphatic drainage disruption was elevated ICPs, which could lead to a cascade of worsening ICPs given the decreased ISF drainage caused by the disruption of meningeal lymphatics.^23^ It is thought that because lymphatics are devoid of smooth muscle, they are especially susceptible to elevation in ICP. Bolte et al^23^ also investigated the role of improving lymphatic drainage and introduced vascular endothelial growth factor-C using a viral vector, which increases meningeal lymphatic vessel size, and found a decrease in TBI-related gliosis, indicating that improvement in meningeal lymphatic drainage may improve TBI outcomes. Although these findings are new, they suggest a possible future therapy in which increases in meningeal-based lymphatic drainage may be used to modify long-term TBI outcomes.

## SUBARACHNOID HEMORRHAGE

Another area in which the discovery of the glymphatic system is altering our understanding of disease is in patients with subarachnoid hemorrhage (SAH). SAH occurs when there is blood within the subarachnoid space, and although this can be caused by trauma, SAH from aneurysm rupture is most commonly examined with the glymphatic system.^24,25^ The interest in SAH caused by vascular etiologies comes from the significant morbidity associated with SAH in both the acute and delayed period.^26^ One of the major causes of morbidity and mortality after SAH is delayed cerebral ischemia, which occurs days to weeks after SAH (usually aneurysmal) and reviewed extensively by Dodd et al.^26^ Another major complication from SAH is posthemorrhagic hydrocephalus which was thought to be caused by obstruction of the arachnoid granulations^27^; however recent evidence suggests that post-SAH hydrocephalus is likely related to another mechanism.

Gaberel et al^11^ introduced SAH and observed a significant decrease in the CSF resorptive capabilities of the glymphatic system using MRI to observe the glymphatic system in living animals. This continued even after bilateral hemicraniectomy, which suggested that it was not due to increased ICP but was instead likely due to direct blockage of the glymphatic system by fibrin.^11^ Another group redemonstrated the same findings but in nonhuman primates^28^ and showed that the fibrin deposition does not just extend to areas in which blood was clearly observed but that fibrin deposits throughout the entire glymphatic system.^29^ Gaberel et al^11^ then injected tPA into the subarachnoid space and found that it broke down the observed fibrin deposits with findings of improvement in CSF flow. Together, these findings suggest that post SAH hydrocephalus is likely caused by deposition of fibrin along the glymphatic system leading to decreases in resorption of CSF and subsequent hydrocephalus.

Although the glymphatic system is critically important in CSF dynamics, the meningeal lymphatics also play an important role after SAH. Meningeal lymphatics were found to clear erythrocytes from the CSF after SAH in rodents.^30^ When meningeal lymphatics are diminished, there is a decrease in the erythrocytes found in cervical lymphatics and increase in neuroinflammation, suggesting that the meningeal lymphatics are likely critical in draining CSF erythrocytes and decreasing the associated inflammatory response from SAH.^30^

Another critical area of research is the role of water channels in CSF production, absorption, and dynamics post SAH. As mentioned earlier, AQP4 has a key role in mixing the CSF with ISF and has an important role in forming the glymphatic system.^10^ In rodents lacking AQP4, there was a decrease in the flow of blood components into the brain parenchyma, but there was no improvement in neurological outcomes, suggesting again that AQP4 is important in glymphatic system but that even reducing blood components into the brain does not improve neurological outcomes from SAH.^31^ Later studies performed by the same group demonstrated that with knockout of AQP4, there was again a decrease in ISF accumulation of blood components but that there was actually a decrease in outflow of neurotoxic components as well, leading to prolonged neurological deficits in AQP4 knock-out mice.^32^ Interestingly, this study also found in the sham group an increase in AQP4 expression along the arteries after SAH, but no change in the AQP4 expression around veins.^32^ Coupled with previous findings suggestive of increasing AQP4 expression on astrocytes post-SAH,^27^ this would be a possible explanation for edema post-SAH: increasing ISF because of increased AQP4 expression at astrocyte endplates but no change in AQP4 expression along venous channels to absorb the ISF into the blood. Therefore, the role of AQP4 after SAH is complicated, leading to increased edema within brain parenchyma but eventually leading to appropriate clearance of neurotoxic blood components out of the brain and CSF spaces.

## NORMAL PRESSURE HYDROCEPHALUS

The role of the glymphatic system and dementia is also of significant interest and of most relevance is normal pressure hydrocephalus (NPH). NPH is a relatively common cause of dementia affecting 10–22 of 100 000 people and is characterized by impaired gait, urinary incontinence, and dementia.^33^ These patients are typically seen with ventriculomegaly but without elevated ICP. The exact etiology of NPH is unknown, but many of these patients improve with CSF diversion.^33^ A study performed in 2017 examined the glymphatic system of patients with NPH (15 patients) and compared them with patients undergoing MRI for CSF leak (7). In all patients, contrast injected into the lumbar space was seen traveling along Virchow-Robin spaces and into the brain along the glymphatics.^34^ However, in patients with NPH, there was increased reflux of contrast into the ventricles, transependymal contrast enhancement, and delay in enhancement of the brain and clearance compared with the controls.^34^ These findings were replicated using 30 patients with NPH and 8 control patients; although this group focused on the entorhinal cortex and found decreased tracer clearance from the entorhinal cortex in the patients with NPH.^35^ Given the importance of the entorhinal cortex in memory, it was suggested that delayed clearance may lead to dementia-like symptoms in those patients because of buildup of neurotoxins. Together, both studies suggest that a possible etiology of NPH may be due to impairment in the glymphatic system, leading to build up of CSF and possible accumulation of parenchymal neurotoxins.

A well-known risk factor for developing dementia is hypertension; although traditionally thought to be related to vascular dementia, there appears to be a relationship to NPH as well.^36,37^ Using microspheres in the brains of rodents with cranial windows, Mestre et al^38^ found that there was a significant decrease in CSF flow through Virchow-Robin spaces into the brain of rodents with hypertension. It is hypothesized that the etiology of this decrease in CSF flow is related to a loss in pulsatility seen in the arteries with hypertension. This decrease in CSF flow into the brain parenchyma may lead to impaired CSF drainage and buildup of CSF within the ventricles, cisterns, and subarachnoid spaces as seen in patients with NPH.

AQP4 also appears to play a role in NPH and the glymphatic system. When comparing the localization of AQP4 in postmortem patients with those of patient's without NPH, there was a significant change in expression of AQP4 in patients with a diagnosis of NPH.^39^ There was lower density of AQP4 along the astrocytic footplates of glia facing capillaries in patients with NPH compared with controls. This decrease in AQP4 could lead to a decrease in the ability to clear interstitial fluid and may be one of the causes of impaired CSF flow.

Although there is still much to be studied in the role of the glymphatic system and NPH, there does appear to be a significant connection between glymphatic system disturbances and the development of NPH. Further studies of the connection between the 2 may lead to additional or new therapeutic intervention.

## ONCOLOGY

The role of the glymphatic system in brain oncological processes is even more in its infancy than those listed above. Using techniques to noninvasively image perivascular spaces, Toh et al found that peritumoral edema in gliomas, metastatic lesions, and meningiomas were associated with impairment in the glymphatic system.^40-42^ It was previously thought that peritumoral edema was associated with microvascular proliferation; but in the previously mentioned studies, there was no associated cerebral blood flow and edema^41^ which supports a previous study using markers for microvascular density that also found no correlation with microvascular density and associated peritumor edema in metastatic melanoma.^43^ Interestingly, there is a strong correlation with peritumoral edema and upregulation of AQP4^44^ which may suggest that at least part of the associated edema in oncological lesions of the brain is associated with dysfunction of the glymphatic system, but this requires further study.

How metastatic lesions seed themselves in brain parenchyma is another area of active research with evidence suggesting a complex interplay between the vasculature and CSF.^45,46^ Although direct evidence implicating the glymphatic system is limited at present, processes such as leptomeningeal metastases likely involve the glymphatic system in their pathogenesis and subsequent development of hydrocephalus.^46^ Although the role of the glymphatic system in oncological processes is still in its infancy, the glymphatic system appears to be potentially implicated in primary and metastatic lesions of the brain and rife for further study.

## CONCLUSION

The glymphatic system, although somewhat recently discovered, appears to have great clinical significance in a multitude of clinical conditions from TBI to dementia.

One area that appears promising is the localization of AQP4. Given the importance of AQP4 in the glymphatic system, controlling its localization may lead to improved CSF drainage and could be a potential target for treating patients with TBI, post-SAH hydrocephalus, and NPH. However, to use AQP4 will require significant work to further understand how AQP4 is polarized, how that polarization is altered in pathological states, and how to repolarize AQP4 before it can be effectively harnessed in a therapeutic capacity.

There is also great promise in the understanding and treatment of TBI. Although direct evidence is currently limited in humans, suggestions of worsening glymphatic and meningeal lymphatic drainage in the setting of elevated ICPs in animal models could mean that a more aggressive approach to ICP management may prevent a cascade of elevated ICPs. Cisternostomy, which is still in its infancy as a treatment for TBI and requires significantly more research to determine safety and efficacy, may be an important treatment in the arsenal of neurosurgeons moving forward to potentially mitigate brain parenchymal edema, preventing brain herniation, and avoiding associated secondary injury that may occur with brain herniation out of a craniectomy defect.

In the field of oncology, further understanding of the role of the glymphatic system in metastasis portends opportunities for further research and potential therapies. Although there is still much to learn about the glymphatic system, further understanding will likely lead to new and, hopefully, better therapeutic options for those with these neurological diseases.

## COMMENTS

This article is a very timely review of some of the key clinical situations wherein disorders of CSF egress and interstitial fluid flow are aberrant. The clinical scenarios discussed that involve neurosurgeons include normal pressure hydrocephalus (NPH), traumatic brain injury (TBI), aneurysmal subarachnoid hemorrhage (aSAH), and brain tumors, to name just a few. The authors nicely review these situations and present data that implicate abnormal flow of fluid through the brain whether it is via the glymphatics or the traditional CSF pathways. Many of the procedures that we have been doing for years as neurosurgeons, unbeknownst to us, achieve their desired effect(s) via modulation of the glymphatic system. For example, consider the chronic SDH, one of the most common problems we face as neurosurgeons. These are associated with increased atrophy and dementia.^1a^ The simple drainage of the cSDH with fenestration of the inner membrane allows the brain to re-expand and re-establish the arachnoid-dural contacts and thus drainage via the dural lymphatics.^2a^

Glymphatic function is an area of intense recent research. Normal glymphatic function is important for the clearance of protein waste products and the prevention of their accumulation. The classic protein currently being examined is beta amyloid and its deposition in Alzheimer disease. Recent efforts to manipulate the glymphatic flow via focused ultrasound to help in the clearance of proteins are intriguing.^3a,4a^ As neurosurgeons, we have the unique opportunity to modulate the glymphatic/dural lymphatic flow and function using neurosurgical techniques such as shunts or cisternostomy. A better understanding of the molecular and physiologic underpinnings of normal CSF dynamics can help us to better modify our current techniques to optimize the outcome for our patients. We will also undoubtedly be called upon to assist with clinical trials directed at the glymphatics. A better understanding of the glymphatic-dural lymphatics will help us to more intelligently assist in these endeavors.

**Jeff W. Chen**

*Irvine, California, USA*
